# Ino80 promotes cervical cancer tumorigenesis by activating Nanog expression

**DOI:** 10.18632/oncotarget.12667

**Published:** 2016-10-14

**Authors:** Jing Hu, Jie Liu, Aozheng Chen, Jia Lyu, Guihai Ai, Qiongjing Zeng, Yi Sun, Chunxia Chen, Jinbo Wang, Jin Qiu, Yi Wu, Jiajing Cheng, Xiujuan Shi, Liwen Song

**Affiliations:** ^1^ Department of Obstetrics and Gynecology, Shanghai Tenth People's Hospital, Tongji University School of Medicine, Shanghai, China; ^2^ Shanghai Tenth People's Hospital, Tongji University School of Medicine, Shanghai, China; ^3^ The First Clinical Medical College of Nanjing Medical University, Nanjing, Jiangsu, China

**Keywords:** Ino80, Nanog, cervical cancer, tumorigenesis, proliferation

## Abstract

Ino80 ATPase is an integral component of the INO80 ATP-dependent chromatin-remodeling complex, which regulates transcription, DNA repair and replication. We found that Ino80 was highly expressed in cervical cancer cell lines and tumor samples. Ino80 knockdown inhibited cervical cancer cell proliferation, induced G0/G1 phase cell cycle arrest *in vitro* and suppressed tumor growth *in vivo*. However, Ino80 knockdown did not affect cell apoptosis, migration or invasion *in vitro*. Ino80 overexpression promoted proliferation in the H8 immortalized cervical epithelial cell line, which has low endogenous Ino80 expression as compared to cervical cancer cell lines. Ino80 bound to the Nanog transcription start site (TSS) and enhanced its expression in cervical cancer cells. Nanog overexpression in Ino80 knockdown cell lines promoted cell proliferation. This study demonstrated for the first time that Ino80 was upregulated in cervical cancer and promoted cell proliferation and tumorigenesis. Our findings suggest that Ino80 may be a potential therapeutic target for the treatment of cervical cancer.

## INTRODUCTION

Incidences of cervical cancer in developed countries have decreased dramatically because of cytologic screening and DNA testing for high-risk human papillomavirus. However, cervical cancer remains the fourth most common cancer in females worldwide, with approximately 527,600 new cases and 265,700 deaths annually, and nearly 90% of deaths occurring in developing countries [1]. Although cures are achieved at early stages with radical surgery, chemo-radiotherapy or both, there are limited options for advanced (recurrent, persistent or metastatic) cervical cancers [2–6]. The 5-year survival rate for women with advanced disease is only 16% [7].

Aberrations in chromatin regulators, such as in histone-modifying enzymes and chromatin remodelers, are associated with diverse cancers [8–11]. Mutations in genes encoding subunits of the SWI/SNF chromatin remodeling complexes are found collectively in 20% of all human cancers, approaching the frequency of p53 mutations [9]. Genetic variations in DNA- and histone-modifying genes are novel predictive biomarkers of recurrence and survival in early stage non-small cell lung cancer patients [8].

The ATP-dependent chromatin remodeling complexes can utilize the energy of ATP hydrolysis to modulate chromatin structure, and are critically involved in processes that require DNA access such as transcription, replication and repair [12, 13]. All of these complexes include a sucrose non-fermenting 2 (SNF2) family ATPase with ATP-dependent nucleosome remodeling activity [14]. The Ino80 ATPase is a member of the SNF2 family ATPases and is an integral component of the INO80 ATP-dependent chromatin remodeling complex (INO80) [15]. Recent studies reveal that INO80 is involved in DNA and telomere replication, DNA repair, transcription regulation and maintenance of genome stability [16–22]. INO80 dysfunction may perturb DNA synthesis, gene regulation and DNA repair, potentially leading to genome instability and the development of cancer. A recent study demonstrates that Ino80 interacts with BRCA1-associated protein-1 (BAP1), a tumor suppressor that also stabilizes Ino80, in normal DNA replication [23]. Ino80 is downregulated in BAP1-defective cancer cells due to destabilization, suggesting a molecular basis for the BAP1 tumor-suppressor function. Another study reports that Ino80 is required for efficient cell proliferation, and *Ino80*^+/−^*p53*^−/−^ mice exhibit a striking shift from lymphomas to sarcomas compared to *p53*^−/−^ mice [19]. This suggests that Ino80 influences tumor type.

Pluripotent transcription factors, which help maintain embryonic stem cell (ESC) self-renewal and pluripotency, are involved in tumorigenesis and progression in various cancers, including cervical cancer [24–28]. For example, Nanog knockdown by small interfering RNA (siRNA) reduces cell proliferation and induces G0/G1 cell cycle arrest in breast cancer cells [28]. High SOX2 and OCT4 expression indicates radiation resistance and poor prognosis in cervical cancer patients [27]. Oct4 directly induces miR-125b expression, inhibiting its direct target BAK1, and suppressing cervical cancer cell apoptosis [26].

Interactions between chromatin regulators and pluripotent transcription factors are essential for ESC self-renewal and pluripotency. Tryptophan aspartic acid (WD) repeat domain 5 (WDR5), an H3K4 methylation effector, reportedly mediates self-renewal and reprogramming via binding with OCT4 in ESCs [29]. INO80 facilitates pluripotency gene activation in ESCs by binding OCT4 and WDR5 [30]. However, little is known about their interactions in cancer cells. In this study, we investigated the role of Ino80 in cervical cancer tumorigenesis, along with the related genetic and epigenetic regulatory mechanisms.

## RESULTS

### Ino80 is highly expressed in cervical cancer

**Figure 1:**
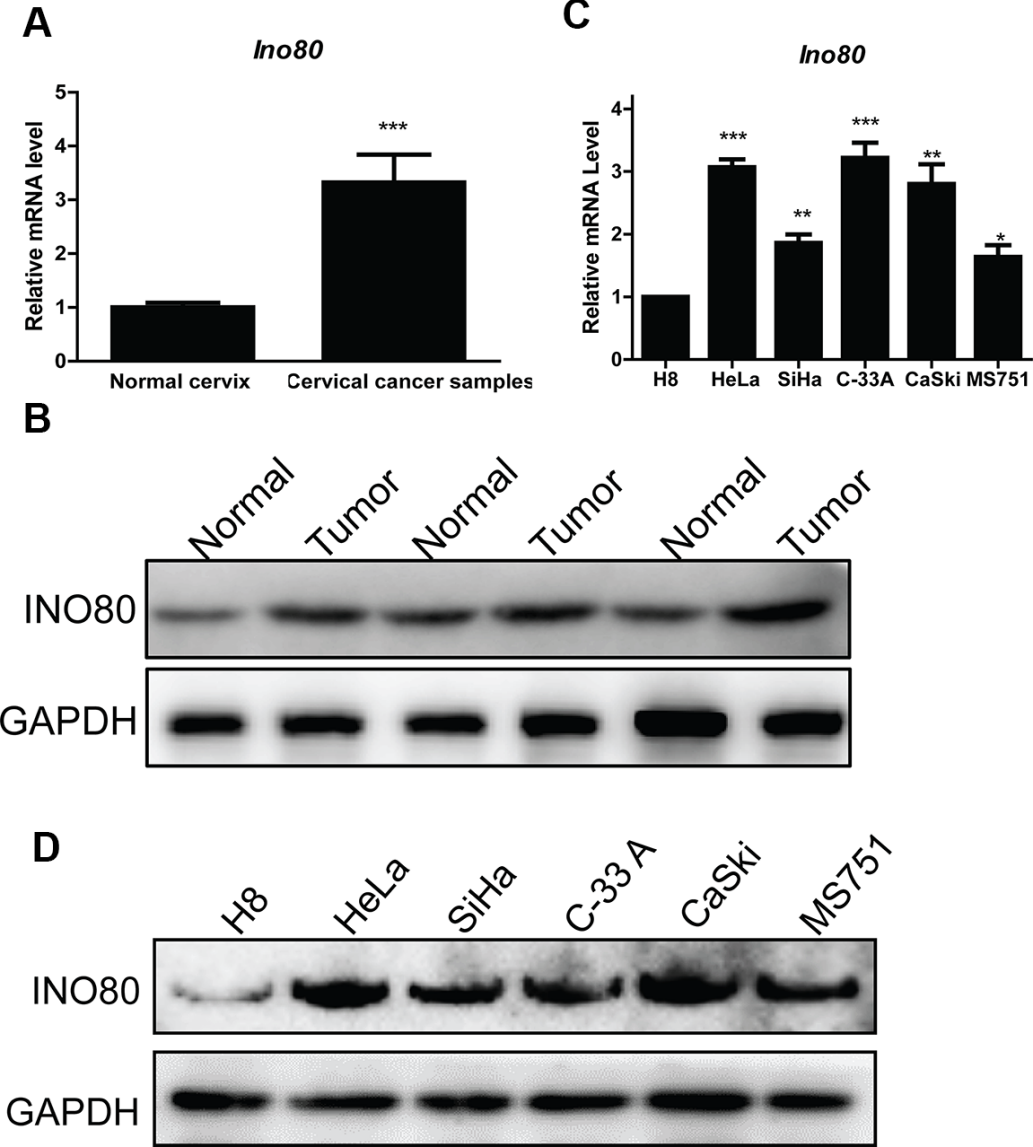
Ino80 is highly expressed in cervical cancer Ino80 expression in normal cervical tissues and cervical cancer samples from one published microarray gene expression dataset (GEO Accession number: GSE9750) (**A**). Data are displayed relative to normal cervix group as means ± SEM. ***p < 0.001. Ino80 expression in cervical cancers (tumor) and corresponding pericarcinous tissues (normal) from three patients (**B**). qRT-PCR analysis of Ino80 in the human immortalized cervical epithelial cell (H8) and cervical cancer cell lines (HeLa, SiHa, C-33A, CaSki and MS751) (**C**). Data are displayed relative to H8 as means ± SEM (*n* = 3). *p < 0.05, **p < 0.01, ***p < 0.001. Western blot analysis of Ino80 in H8, HeLa, SiHa, C-33A, CaSki and MS751 cells with GAPDH as a loading control (**D**).

To assess Ino80 expression in cervical cancer, we examined gene expression data from one published study consisting of 9 cervical cancer cell lines, 24 normal cervical tissues and 28 cervical cancer samples [31]. Compared to normal cervical tissues, Ino80 was markedly upregulated in cervical cancer samples (Figure 1A). Ino80 expression was higher in cervical cancers as compared to corresponding pericarcinous tissues (Figure 1B). Similarly, Ino80 expression was elevated in the human cervical cancer cell lines, HeLa, SiHa, C-33A, CaSki and MS751, compared to the immortalized cervical epithelial cell line, H8 (Figure 1C–1D).

### Ino80 knockdown does not influence cervical cancer cell apoptosis

**Figure 2:**
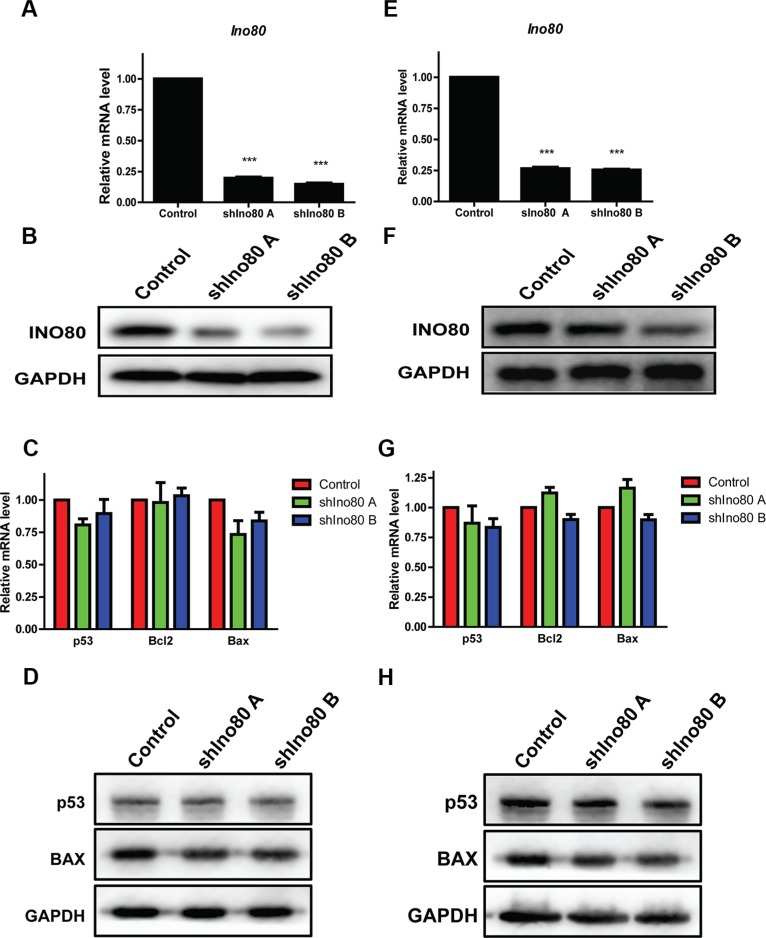
Ino80 knockdown and cell apoptosis detection qRT-PCR analysis of Ino80 in control (scrambled shRNA) and Ino80 knockdown HeLa (**A**) and SiHa (**E**) cells. Western blot analysis of Ino80 in control and Ino80 knockdown HeLa (**B**) and SiHa (**F**) cells. qRT-PCR analysis of p53, Bcl2 and Bax in control and Ino80 knockdown HeLa (**C**) and SiHa (**G**) cells. Western blot analysis of p53 and BAX in control and Ino80 knockdown HeLa (**D**) and SiHa (**H**) cells. Western blots used GAPDH as a loading control. qRT-PCR data are displayed relative to controls as means ± SEM (*n* = 3). ***p < 0.001.

We designed two shRNAs targeting Ino80: shIno80 A and shIno80 B. All shRNA-infected HeLa and SiHa cells expressed ZsGreen under fluorescence microscopy observation (Figure S1A–S1B). Compared with the control (scrambled shRNA), shIno80 A and B efficiently downregulated Ino80 mRNA (Figure 2A and 2E) and protein (Figure 2B and 2F) in both cell lines.

The INO80 chromatin complex is critically involved in DNA repair. Therefore, we examined whether Ino80 knockdown affected cervical cancer cell apoptosis. p53, Bcl2 and Bax mRNA and protein levels were unchanged following Ino80 knockdown as detected by qRT-PCR and western blot analyses (Figure 2C–2D and 2G–2H) and this was confirmed by TUNEL assay (Figure S1C–S1D). These data demonstrate that Ino80 knockdown does not impact cervical cancer cell apoptosis.

### Ino80 knockdown inhibits cervical cancer cell proliferation

**Figure 3:**
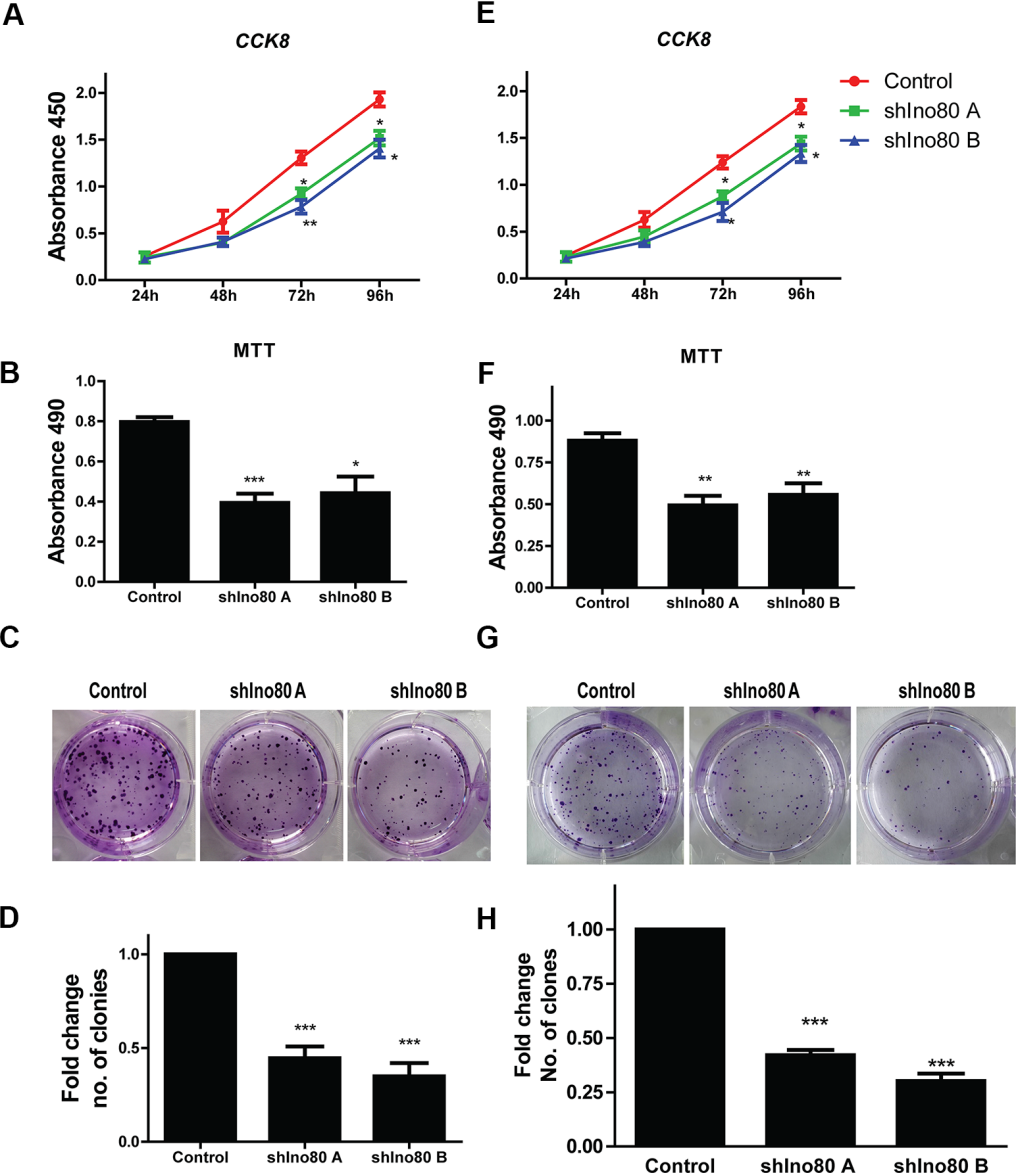
Ino80 knockdown inhibits cell proliferation Growth curves of control and Ino80 knockdown HeLa (**A**) and SiHa (**E**) cells constructed from CCK-8 assay results. Influence of Ino80 knockdown on HeLa (**B**) and SiHa (**F**) cell viability as measured by MTT assay 48 h post-cell seeding. The data in CCK-8 and MTT assays are represented as means ± SEM (*n* = 3). *p < 0.05, **p < 0.01, ***p < 0.001. Representative images of colonies formed by control and Ino80 knockdown HeLa (**C**) and SiHa (**G**) cells, stained with crystal violet. Fold change in number of colonies formed by HeLa (**D**) and SiHa (**H**) cells. Data are represented relative to controls as means ± SEM (*n* = 3). ***p < 0.001.

Ino80 was required for efficient mouse embryo fibroblast (MEF) proliferation [19]. We hypothesized that Ino80 may also promote cervical cancer cell proliferation. Ino80 knockdown inhibited HeLa and SiHa cell proliferation as measured via CCK-8 assay (Figure 3A and 3E). Colony formation assay showed that Ino80 knockdown in these cells decreased the number of formed colonies (Figures 3C–3D and 3G–3H). Ino80 knockdown also reduced cell viability as detected by MTT assay (Figure 3B and 3F). These data suggest that Ino80 promotes cervical cancer cell proliferation *in vitro*.

### Ino80 knockdown induces G0/G1 phase cell cycle arrest

**Figure 4:**
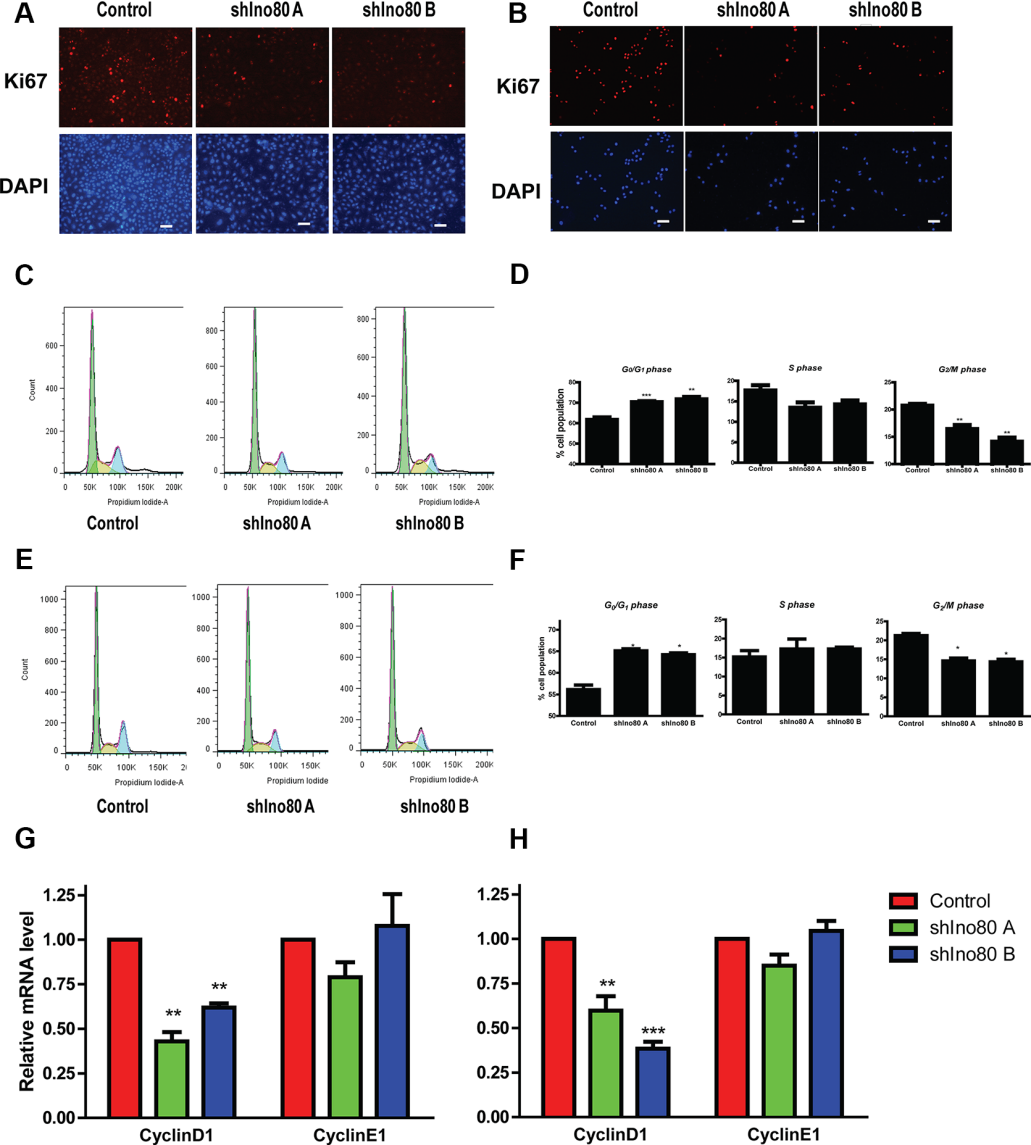
Ino80 knockdown induces G0/G1 phase cell cycle arrest Ki67 expression in control and Ino80 knockdown HeLa (**A**) and SiHa (**B**) cells as determined by IF staining. Nuclei were counterstained with DAPI. Bars = 40 μm. Effect of In80 knockdown on cell cycle progression in HeLa (**C**) and SiHa (**E**) cells. FACS data were analyzed by ModFit LT software. Histograms show the percentage (%) of HeLa (**D**) and SiHa (**F**) cell populations at different cell cycle stages. Data are represented as means ± SEM (*n* = 3). *p < 0.05, **p < 0.01, ***p < 0.001. qRT-PCR analysis of cyclinD1 and cyclinE1 in control and Ino80 knockdown HeLa (**G**) and SiHa (**H**) cells. Data are represented relative to controls as means ± SEM (*n* = 3). **p < 0.01, ***p < 0.001.

Ki67 protein is a proliferation marker present during all active phases of the cell cycle (G1, S, G2 and mitosis), but absent from resting cells (G0). Ino80 knockdown in HeLa and SiHa cells resulted in lower Ki67 expression compared with controls (Figure 4A–4B). We used flow cytometry to determine whether Ino80 was involved in cervical cancer cell cycle regulation. Ino80 knockdown in HeLa and SiHa cells dramatically increased the cell population at G0/G1 phase, and reduced the cell population at S and G2/M phases (Figure 4C–4F). CyclinD1 expression also decreased in Ino80 knockdown cells (Figure 4G–4H). Collectively, these results demonstrate that Ino80 knockdown mainly induces G0/G1 phase cell cycle arrest in cervical cancer cells.

### Ino80 knockdown does not affect cervical cancer cell migration and invasion

Wound healing and transwell chamber migration assays suggested Ino80 knockdown in HeLa and SiHa cells did not affect cellular migration compared with controls (Figure S2A–S2B). Similarly, cell invasion through an extracellular matrix did not change following Ino80 knockdown (Figure S2B). Mmp2, Mmp9 and Mmp11 mRNA levels were also unchanged in control and Ino80 knockdown cells (Figure S2C). These data indicate that Ino80 does not regulate cervical cancer cell migration and invasion.

### Ino80 knockdown suppresses cervical cancer cell growth *in vivo*

**Figure 5:**
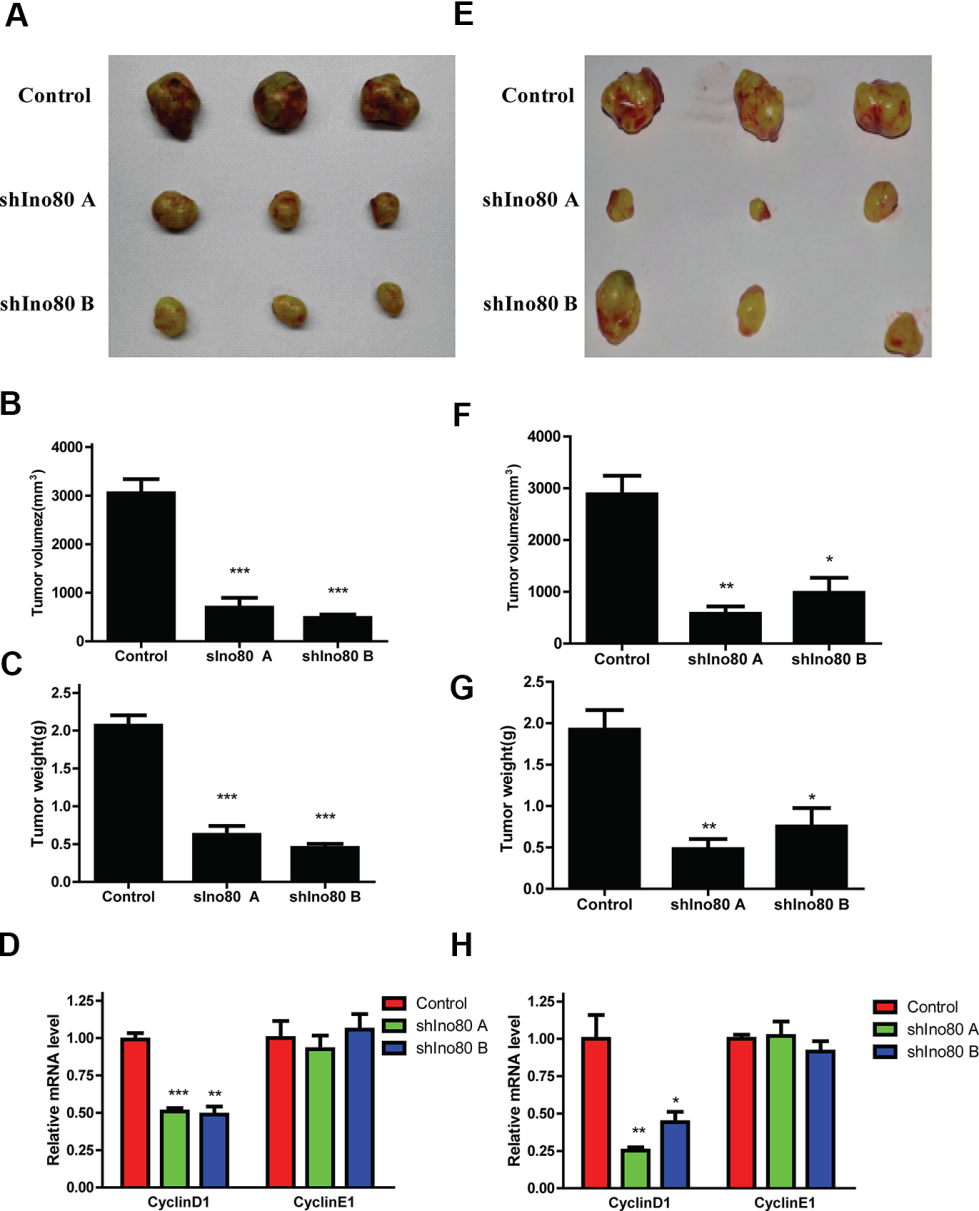
Ino80 knockdown suppresses cervical cancer cell growth *in vivo* Images of tumors derived from control and Ino80 knockdown HeLa (**A**) and SiHa (**E**) cells four weeks post-inoculation. Control and Ino80 knockdown HeLa (**B**) and SiHa (**F**) cell tumor volumes four weeks post-inoculation. Control and Ino80 knockdown HeLa (**C**) and SiHa (**G**) cell tumor weights four weeks post-inoculation. Data are represented as means ± SEM (*n* = 3). *p < 0.05, **p < 0.01, ***p < 0.001. qRT-PCR analysis of cyclinD1 and cyclinE1 in control and Ino80 knockdown HeLa (**D**) and SiHa (**H**) cell groups. Data are represented relative to controls as means ± SEM (*n* = 3). *p < 0.05, **p < 0.01, ***p < 0.001.

Stable Ino80 knockdown or control HeLa and SiHa cells were subcutaneously injected into female nude mice and tumor growth was measured. Growth of tumors derived from Ino80 knockdown cells was suppressed compared with controls four weeks post-inoculation (Figure 5A and 5E). Ino80 knockdown group tumor volumes and weights were reduced compared to controls (Figure 5B–5C and 5F–5G). CyclinD1 expression was also decreased in Ino80 knockdown groups (Figure 5D and 5H). These results indicate that Ino80 promotes cervical cancer cell growth *in vivo.*

### Ino80 overexpression promotes cervical epithelial cell proliferation

**Figure 6:**
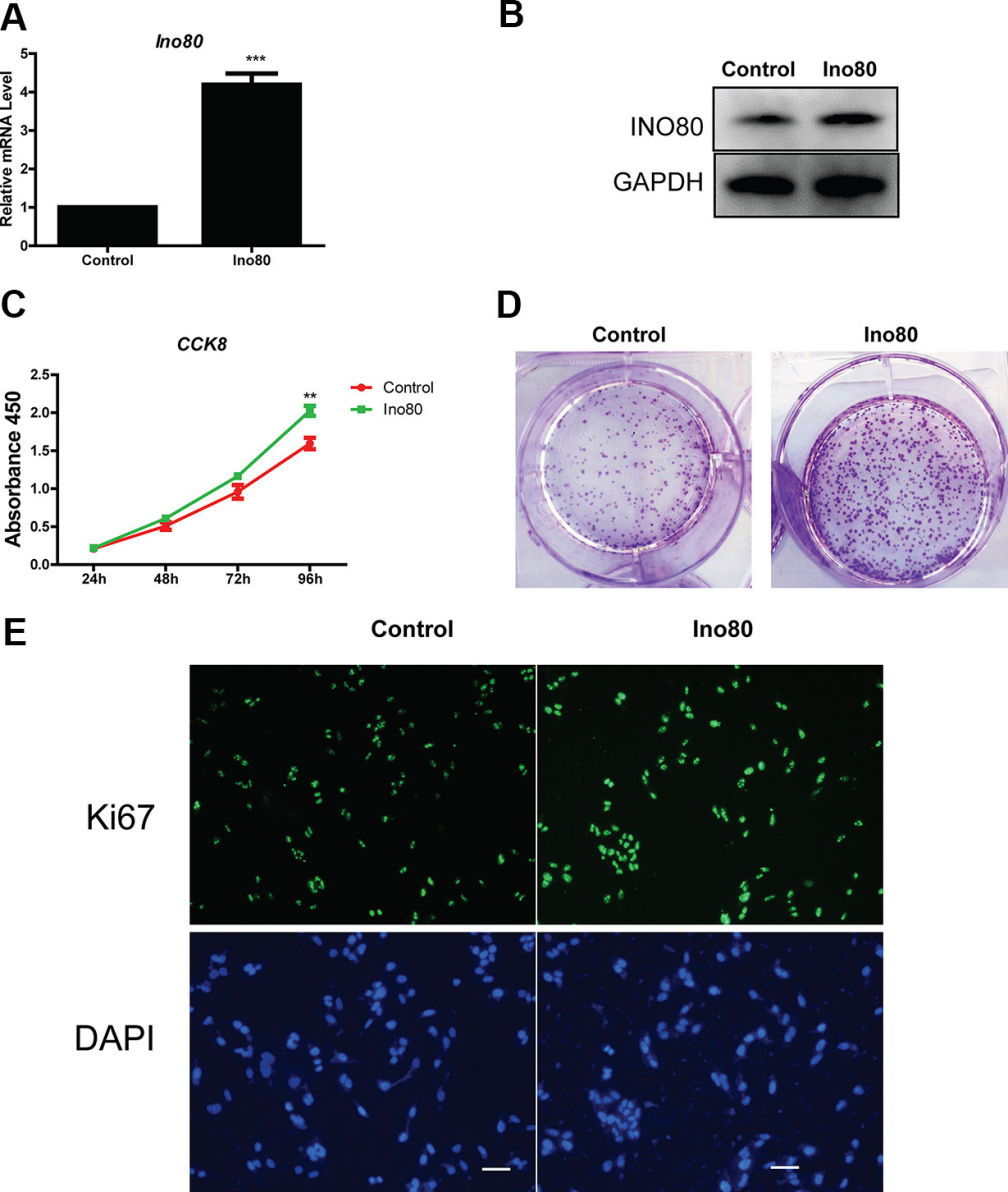
Ino80 overexpression promotes cervical epithelial cell proliferation qRT-PCR (**A)** and western blot (**B**) analyses of Ino80 in control and Ino80 overexpression H8 cells. qRT-PCR data are represented relative to control as mean ± SEM (*n* = 3). ***p < 0.001. Western blot analysis used GAPDH as a loading control. Control and Ino80 overexpression H8 cell growth curves constructed using CCK-8 assay results (**C**) Data are represented as means ± SEM (*n* = 3). **p < 0.01. Colony formation in control and Ino80 overexpression H8 cells (**D**) Colonies were stained with crystal violet. Ki67 expression in control and Ino80 overexpression H8 cells as determined by IF staining (**E**) Nuclei were counterstained with DAPI. Bars = 40 μm.

We investigated whether Ino80 knockdown inhibited proliferation in the human immortalized cervical epithelial cell line, H8. Ino80 mRNA and protein levels in H8 cells were decreased following Ino80 knockdown (Figure S3A and S3B). CCK8 and colony formation assays showed similar proliferation rates in control and Ino80 knockdown cells (Figure S3C–S3D). Similarly, Ki67 levels were also unchanged (Figure S3E). However, H8 cells express lower levels of endogenous Ino80 than do cervical cancer lines (Figure 1C–1D). Therefore, we overexpressed Ino80 in H8 cells (Figure 6A–6B). Ino80 overexpression promoted H8 cell proliferation as detected by CCK8 and colony formation assays (Figure 6C–6D), and enhanced Ki67 expression (Figure 6E).

### Ino80 promotes Nanog expression by binding its transcription start site

**Figure 7:**
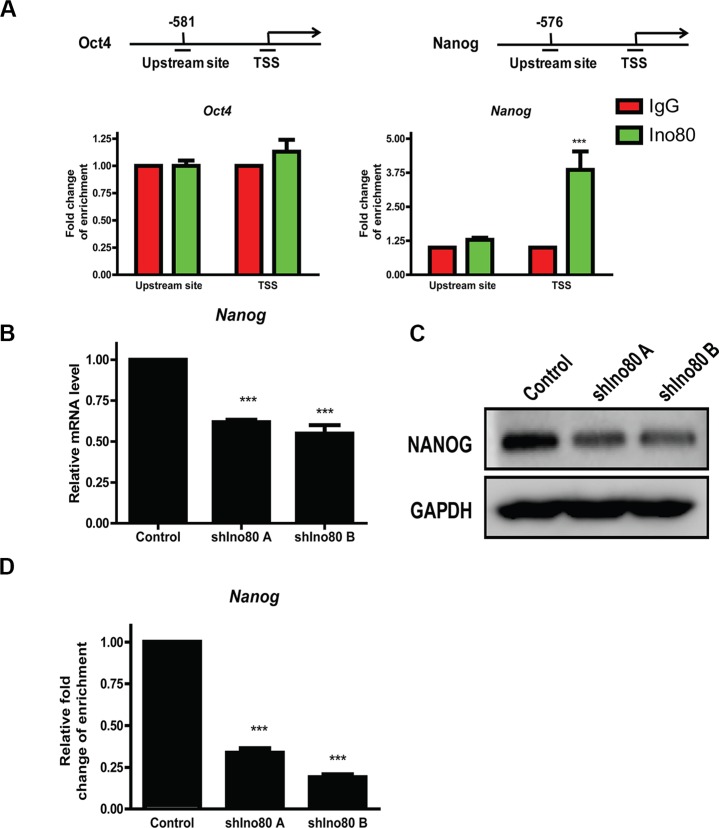
Ino80 promotes Nanog expression by binding to its transcription start site (TSS) ChIP-qPCR analysis showing Ino80 binding levels in the Oct4 and Nanog gene TSS and upstream sites in HeLa cells (**A**) Enrichment fold changes are normalized to IgG binding and is represented as mean±SEM (*n* = 3). ***p < 0.001. qRT-PCR (**B**) and western blotting (**C**) analysis of Nanog in control and Ino80 knockdown HeLa cells. Western blot analysis used GAPDH as a loading control. ChIP-qPCR analysis showing Ino80 binding levels in the Nanog TSS in control and Ino80 knockdown HeLa cells (**D**) Data in B and D are represented relative to controls as means ± SEM (*n* = 3). ***p < 0.001.

Ino80 bound to pluripotency gene promoter proximal regions of Oct4 and Nanog to activate their expression, and Ino80 knockdown decreased expression of these key pluripotency factors in ESCs [30]. Oct4 and Nanog reportedly play pivotal roles in cervical cancer progression [26, 32, 33]. We hypothesized that Ino80 may promote cervical cancer tumorigenesis through elevating expression of these pluripotency factors. We found that Oct4 expression was very low in HeLa cells (data not showed), consistent with a previous study [34]. We measured Ino80 binding to Oct4 and Nanog gene transcription start sites (TSS) and upstream sites via ChIP assay followed by quantitative PCR (ChIP-qPCR) and found that Ino80 was present at Nanog, but not Oct4 TSS (Figure 7A). Consistent with our observations in cervical cancer cells, Nanog knockdown by siRNA reportedly reduced proliferation and induced G0/G1 cell cycle arrest in breast cancer cells [28]. Compared with controls, we found that Ino80 knockdown in HeLa cells reduced Nanog expression (Figure 7B–7C) and decreased Ino80 binding in the Nanog TSS (Figure 7D).

### Nanog overexpression promotes cervical cancer cell proliferation

**Figure 8:**
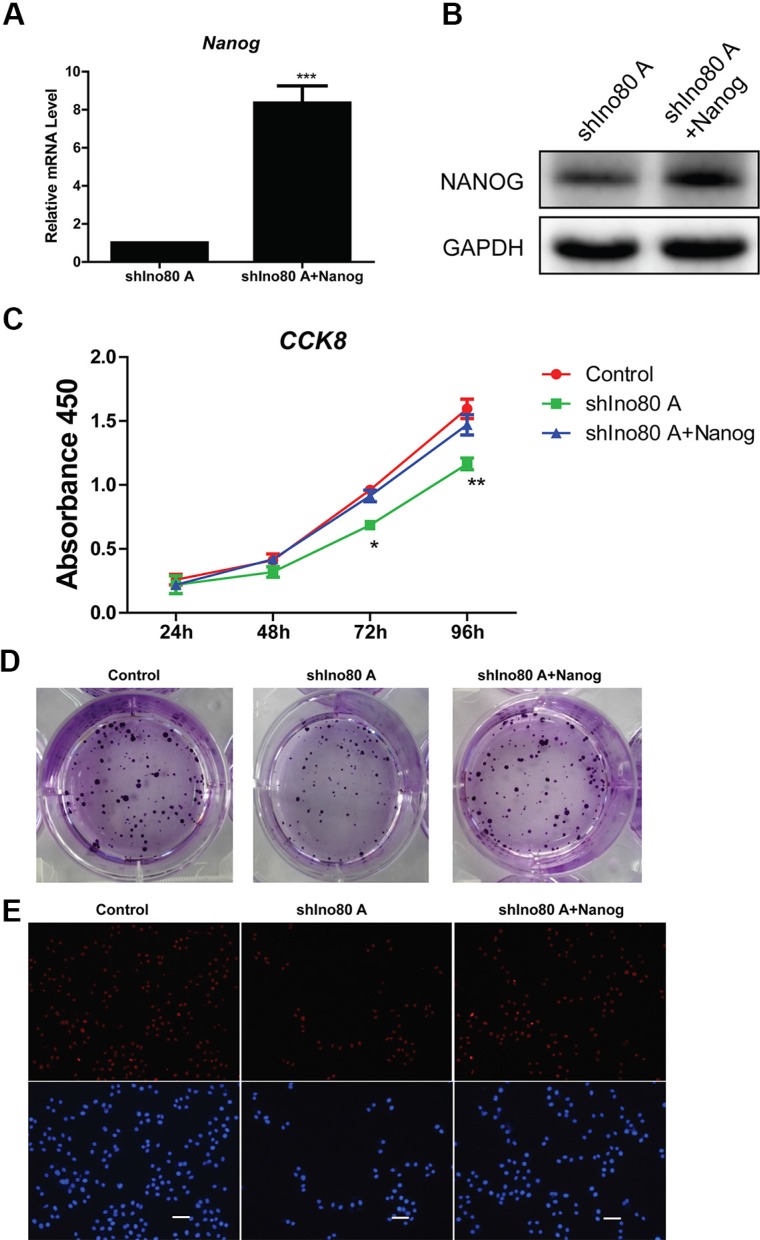
Nanog overexpression promotes cervical cancer cell proliferation Cells are divided into three groups: HeLa cell (control), Ino80 knockdown HeLa cell (shIno80 A) and Nanog overexpression in Ino80 knockdown HeLa cell (shIno80 A + Nanog). qRT-PCR (**A**) and western blotting (**B**) analysis of Nanog in the shIno80 A and shIno80 A + Nanog groups. qRT-PCR data are displayed relative to the shIno80 A group as mean ± SEM (*n* = 3). ***p < 0.001. Western blot analysis used GAPDH as a loading control. Control, shIno80 A and shIno80 A + Nanog HeLa cell growth curves constructed from CCK-8 assay results (**C**). Data are represented as means ± SEM (*n* = 3). *p < 0.05, **p < 0.01. Colony formation in control, shIno80 A and shIno80 A + Nanog HeLa cells (**D**). Colonies were stained with crystal violet. Ki67 expression in control, shIno80 A and shIno80 A + Nanog HeLa cells as determined by IF staining (**E**) Nuclei were counterstained with DAPI. Bars = 40 μm.

We overexpressed Nanog in Ino80 knockdown HeLa cells (Figure 8A–8B). CCK8 and colony formation assays showed that Ino80 knockdown decreased HeLa cell growth rates (Figure 8C–8D). However, Nanog overexpression in Ino80 knockdown HeLa cells promoted cell growth to levels comparable to control (no knockdown) cells (Figure 8C–8D), and elevated Ki67 expression (Figure 8E).

Collectively, these findings indicate that Ino80 binds to the Nanog TSS and enhances its expression in cervical cancer cells to promote tumorigenesis.

## DISCUSSION

Our data demonstrated that Ino80 promoted cervical cancer cell proliferation *in vitro* and tumor growth *in vivo*. Min, *et al.* reported that Ino80 was required for efficient MEF proliferation, and Ino80 deletion reduced proliferation [19]. Wang, *et al.* also reported that Ino80 was required for ESC self-renewal [30]. Our observations are consistent with these reports, and confirm a role for Ino80 in cervical cancer cell proliferation.

Stemness factor Nanog has been identified as a tumorigenic factor and is associated with poor prognosis in many cancer types [35–39]. Nanog was highly expressed in patients with squamous cervical carcinomas [40]. Our data demonstrated that Ino80 bound Nanog transcription start site and activated its expression in cervical cancer cells. We also showed that Nanog overexpression in Ino80 knockdown cervical cancer cells promoted cell proliferation. Our data suggests that Nanog may mediate the pro-proliferative effects of Ino80 in cervical cancer cell.

In this study, we demonstrated for the first time that Ino80 was upregulated in cervical cancer and promoted tumorigenesis. Our findings suggest that Ino80 may be a potential therapeutic target for cervical cancer.

## MATERIALS AND METHODS

### Cell culture

Human cervical cancer cell lines (HeLa, SiHa, C-33A, CaSki and MS751) and a human immortalized cervical epithelial cell line (H8) were cultured in high-glucose Dulbecco's modified Eagle's medium (DMEM) (Gibco, Grand Island, NY) supplemented with 10% fetal bovine serum (FBS) (Gibco) and 1% penicillin/streptomycin (Gibco). Cells were grown in a humidified atmosphere of 5% CO2 at 37°C.

### Patients and specimens

Three pairs of surgically resected cervical cancer and corresponding pericarcinous tissues were obtained from patients without preoperative treatment at Tenth People's Hospital, Tongji University School of Medicine (Shanghai, China) between September 2014 and September 2015. Human specimen collection procedures were approved by the Ethics Committee of Tenth People's Hospital.

### Plasmids and lentivirus constructs

Ino80 shRNA knockdown (shIno80) sequences were obtained from Sigma-Aldrich (Table S1). shIno80 sequences were ligated into the PLVX-shRNA2 vector expressing a classic scrambled shRNA and green fluorescent protein (ZsGreen). Ino80 and Nanog overexpression sequences (Table S2) were ligated into PLVX-IRES-TDTOMATO and FUGW-H1-GFP vectors, respectively. For viral packaging, 293T cells were cotransfected with lentiviral plasmids using the FuGene HD transfection reagent (Roche Diagnostics, Basel, Switzerland). Virus-containing medium was harvested 48 or 72 h post-transfection, then filtered to remove cell debris and used for infection. To generate knockdown or overexpression cell lines, 1 × 10^5^ cells were seeded into six-well plates 1 d before infection. Medium was then replaced with virus-containing supernatant supplemented with 8 μg/ml polybrene (Sigma, St. Louis, MO). Ino80 knockdown and overexpression cells were selected through Flow cytometry using a FACSCalibur system (BD Biosciences). Nanog overexpression cells were selected with G418 (Sigma, St. Louis, MO).

### Cell proliferation assay

Methyl thiazolyl tetrazolium (MTT) assay was used to screen for cell viability. Cells were seeded in 96-well plates at 2 × 10^3^ cells/well. 48 h later, absorbance was measured at a wavelength of 490 nm using a SpectraMax M5 plate reader (Molecular Devices).

For the Cell Counting Kit-8 (CCK8) cell proliferation assay (Dojindo, Kumamoto, Japan), 1 × 10^3^ cells/well were seeded in 96-well plates. After 24, 48, 72 and 96 h, the CCK8 reagent was thawed for approximately 10 min in a water bath at 37°C, and 10 μl of the reagent was added to each well. Plates were incubated at 37°C for 1–4 h. The absorbance at 450 nm was recorded using a SpectraMax M5 plate reader.

For colony formation assay, 500 cells/well were seeded in a 6-well plate. Approximately 14 d later, clones were fixed in 100% methanol and stained with 1% crystal violet in ddH_2_O. Clones were then imaged and quantified.

For cell cycle analysis, cells were fixed in 70% ice cold ethanol followed by RNase A treatment. Cells were stained with 50 μg/ml propidium iodide for DNA content analysis in a BD FACSCalibur flow cytometer. Data were collected using BD FACSuite analysis software and analyzed using ModFit LT software (Verity Software House, Inc).

### Wound healing assay

Culture inserts (ibidi, Martinsried, Germany) consisting of two reservoirs separated by a 500-mm thick wall were placed in a 24-well plate. An equal amount (70 μl) of cell suspension (5 × 10^5^ cells/ml) was added to each reservoir followed by incubation at 37°C. After cell attachment (10 h), culture inserts were gently removed and wells were filled with serum-free culture medium containing 0.2% bovine serum albumin (BSA). The gap between two cell layers was observed under an inverted microscope immediately and after 36 h.

### Transwell assay

Cell migration and invasion were analyzed using transwell chambers (Corning, NY, USA). For migration, HeLa and SiHa cells were suspended in DMEM with 1% FBS and added to the upper chambers (1 × 10^5^ cells/well), which were incubated at 37°C for 24 h. Cells on the upper surface of the membrane were then removed. Membranes were fixed with 4% paraformaldehyde and cells on the undersurface were stained with DAPI. The chambers were observed under a fluorescence microscope. For invasion, chambers were pre-coated with matrigel (BD Biosciences; 50 mg/ml; 1:8) at 37°C for 4 h. After cells were added, chambers were incubated at 37°C for 24 h.

### TUNEL assay

Terminal deoxynucleotidyl transferase dUTP nick end labeling (TUNEL) assay was performed using the one-step TUNEL apoptosis assay kit from Beyotime Institute of Biotechnology in China. Cells treated as indicated were fixed with 4% paraformaldehyde and permeabilized with 0.25% Triton X-100. Cells were incubated with 50 μl TUNEL reaction mixture for 1 h at 37°C in the dark. Cells were observed under a fluorescent microscope (550 nm excitation and 570 nm emission) and images were captured and digitized by image analysis software.

### Immunofluorescence (IF) staining

Cells cultured in 24-well plates were fixed in 4% paraformaldehyde and permeabilized with 0.25% Triton X-100, followed by blocking with 10% FBS in PBS. Cells were then probed with primary anti-Ki67 antibody (Abcam, Cambridge, UK) in 10% FBS overnight at 4°C, then secondary antibody in 10% FBS for 2 h at room temperature. Cells were counterstained with DAPI for 2 min. Cells were washed with and imaged in PBS.

### Western blotting

Cultured cells were lysed in strong RIPA buffer containing Halt Protease Inhibitor Cocktails (Thermo, Waltham, MA). Protein concentrations were measured using a BCA protein assay kit (Pierce, Rockford, IL). Primary antibodies targeting NANOG (Abcam), Ino80 (proteintech, Chicago, IL), OCT4 (Santa Cruz Biotechnology, Santa Cruz, CA), p53 (Santa Cruz Biotechnology), BAX (Abcam) and GAPDH (Santa Cruz Biotechnology) were incubated with the proteins overnight at 4°C, followed by incubation with the appropriate HRP (horseradish peroxidase)-conjugated secondary antibodies. Detection of HRP was performed using the Super Signal West Pico Chemiluminescent Substrate (Pierce).

### Reverse transcription and qRT-PCR

Total RNA was isolated using the Trizol reagent (Invitrogen, Carlsbad, CA) following the manufacturer's instructions. For each sample, 500 ng of RNA was reverse transcribed to cDNA using the Prime-Script RT reagent kit (TaKaRa, Dalian, China). cDNA was amplified with the Takara Ex Taq PCR kit (TaKaRa). qRT-PCR amplification was conducted using the Stratagene Mx3000 QPCR system (Stratagene, Foster City, CA) and analyzed via the ΔΔCT method. Primer sequences are provided in Table S3.

### Chromatin immunoprecipitation assay

Chromatin immunoprecipitation (ChIP) assays were performed with normal rabbit IgG (Millipore, Billerica, MA) and Ino80 (Proteintech) antibody using the EZ ChIP kit (Millipore) according to the manufacturer's protocol. Whole-cell DNA and immunoprecipitated DNA were used for PCR assays with primers targeting sequences surrounding the binding sites. Primer sequences are provided in Table S4. Fold enrichment was calculated relative to normal rabbit IgG.

### Animal studies

All *in vivo* experiments were performed according to approved protocols from the Institutional Animal Care and Use Committee of Tongji University. 5 × 10^6^ HeLa and SiHa cells were injected subcutaneously into the right or left forelimb axillaries of three 4-week-old female nude mice. All mice were killed after four weeks and subcutaneous tumor nodules were extracted and measured.

### Statistical analysis

Error bars represent the SEM of three independent experiments. Data are represented as means ± SEM; *n* = 3. *p* < 0.05 was considered statistically significant (Student's *t* test).

## SUPPLEMENTARY MATERIALS FIGURES AND TABLES


