# Supplementary material for: Ino80 promotes cervical cancer tumorigenesis by activating Nanog expression
Source: Oncotarget. 2016 Oct 14;7(44):72250–62. doi: 10.18632/oncotarget.12667 (PMC5342159; doi:10.18632/oncotarget.12667)
Supplement: Supplementary file 1 [file oncotarget-07-72250-s001.pdf]

Ino80 promotes cervical cancer tumorigenesis by activating Nanog expression

Supplementary Materials

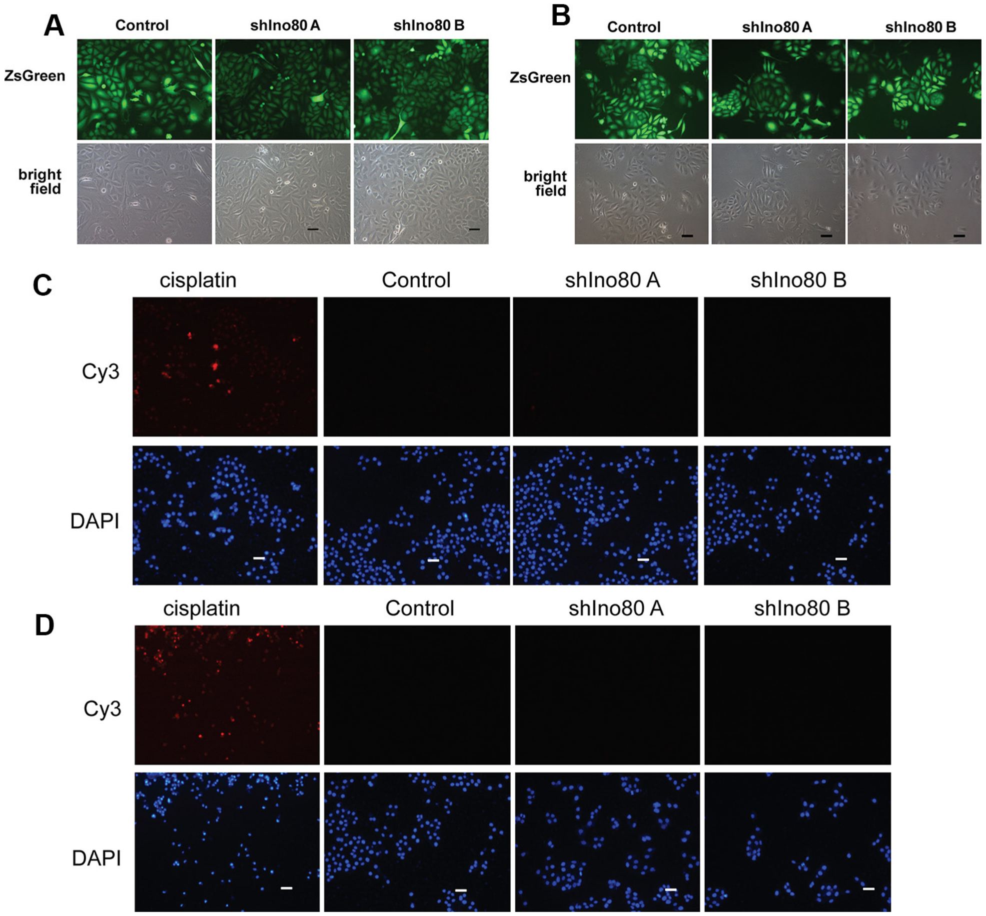

Supplementary Figure S1: Ino80 knockdown and cell apoptosis detection. Morphology of ZsGreen positive lentivirus infected HeLa (**A**) and SiHa (**B**) cells. Bars = 40 μm. TUNEL assay in control and Ino80 knockdown HeLa (**C**) and SiHa (**D**) cells. Images of TUNEL positive cells were captured by a fluorescence microscope. Cells were treated with 5 μg/ml cisplatin for 24 h as positive control.

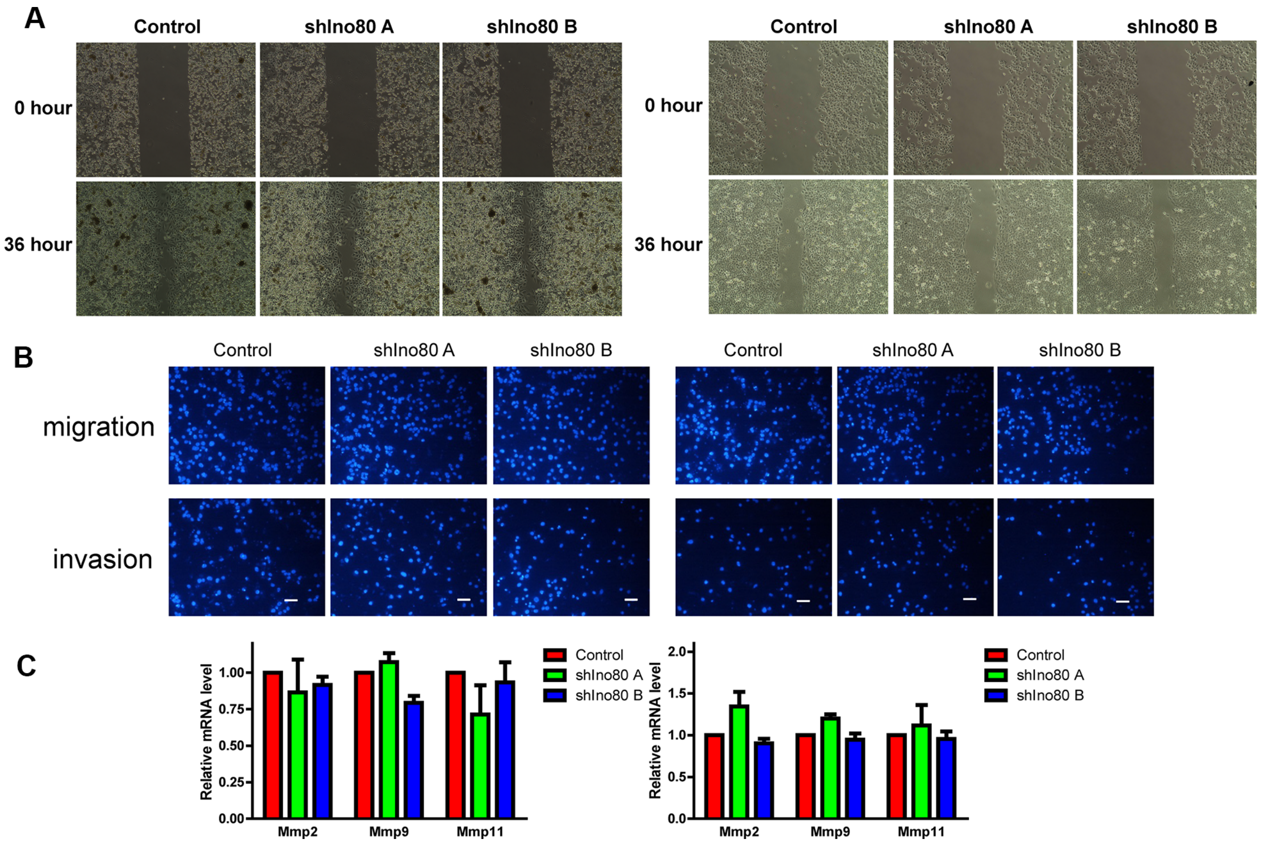

Supplementary Figure S2: Ino80 knockdown does not affect cervical cancer cell migration and invasion. Wound healing assay in control and Ino80 knockdown HeLa (Left) and SiHa (Right) cells (**A**). Transwell chamber migration and invasion assay in control and Ino80 knockdown HeLa (Left) and SiHa (Right) cells (**B**). Representative images of undersurface cells were taken after 24 h incubation. qRT-PCR analysis of Mmp2, Mmp9 and Mmp11 in control and Ino80 knockdown HeLa (Left) and SiHa (Right) cells (**C**). Data are represented relative to controls as means ± SEM (n = 3).

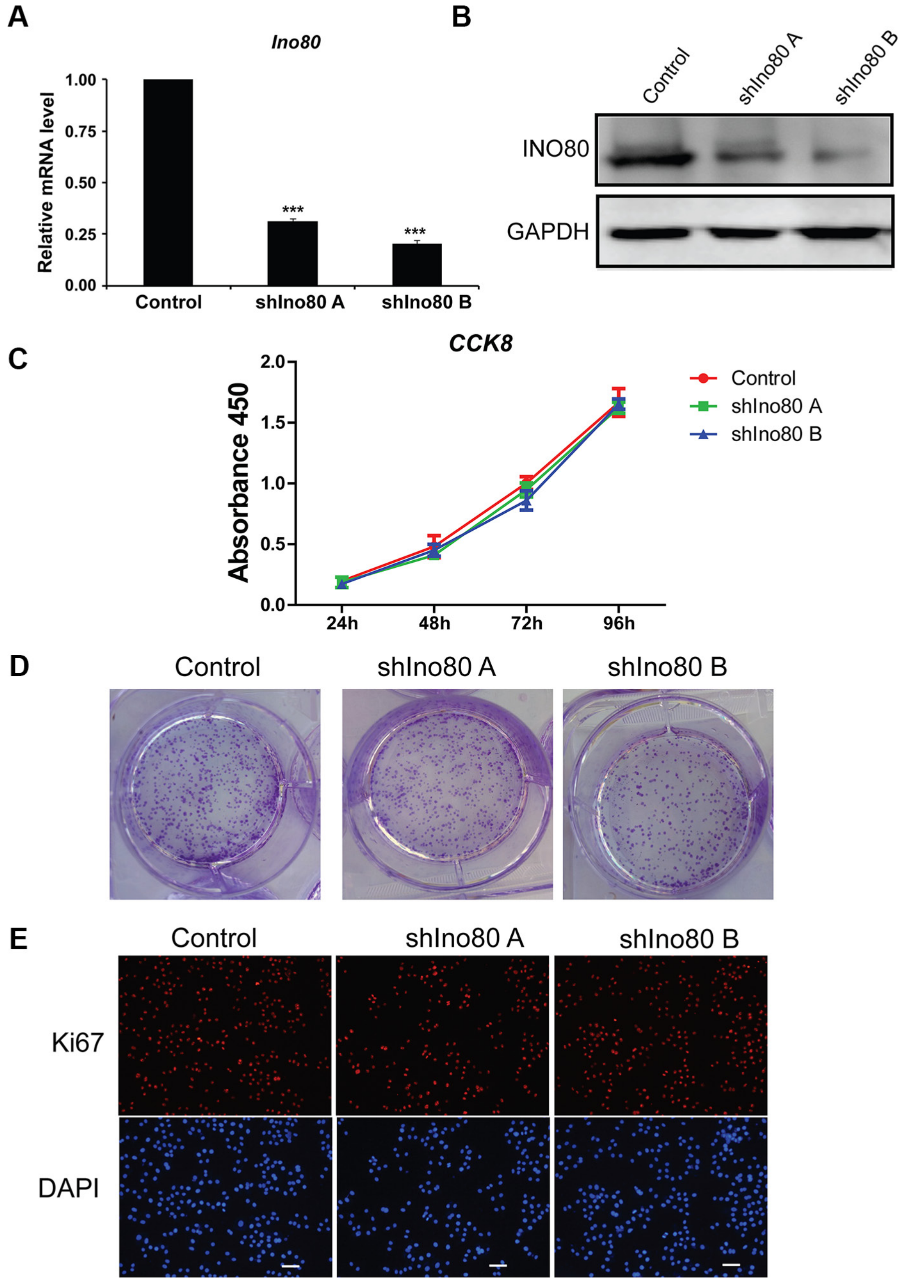

Supplementary Figure S3: Ino80 knockdown does not affect cervical epithelial cell proliferation. qRT-PCR (**A**). and western blotting (**B**). analysis of Ino80 in control and Ino80 knockdown H8 cells. qRT-PCR data are represented relative to control as means ± SEM (n = 3). ***$p < 0.001$.Western blot analysis used GAPDH as a loading control. Control and Ino80 knockdown H8 cell growth curves constructed using CCK-8 assay results (**C**). Data are represented as means ± SEM (n = 3). Colony formation in control and Ino80 knockdown H8 cells (**D**). Colonies were stained with crystal violet. Ki67 expression in control and Ino80 knockdown H8 cells as determined by IF staining (**E**). Nuclei were counterstained with DAPI. Bars = 40 µm.

Supplementary Table S1: The Ino80 shRNA knockdown sequences

Name	Sequences
shscramble-F	gatccCAGCGCTGACAACAGTTTCATCTCGAGATGAAACTGTTGTCAGCGCTGTTTTTg
shscramble-R	aattcAAAAACAGCGCTGACAACAGTTTCATATATCTCATGAAACTGTTGTCAGCGCTGg
shIno80 A-F	gatccCATGAGTCGCAAACGAGATATCTCGAGATATCTCGTTTGCGACTCATGTTTTTg
shIno80 A-R	aattcAAAAACATGAGTCGCAAACGAGATATCTCGAGATATCTCGTTTGCGACTCATGg
shIno80 B-F	gatccGGATGACAGTAATCCATTATTCTCGAGAATAATGGATTACTGTCATCCTTTTTg
shIno80 B-R	aattcAAAAAGGATGACAGTAATCCATTATTCTCGAGAATAATGGATTACTGTCATCCg

Supplementary Table S2: The Ino80 and Nanog overexpression PCR primer

Name	Sequences
Nanog-F	CGCGAATTCATGAGTGTGGATCCAGCTTGTCCC
Nanog-R	CGCGAATTCTCACACGTCTTCAGGTTGCATGT
Ino80-F	CGCGAATTCATGGCCTCGGAGTTGGGTGCCAG
Ino80-R	CGCACTAGTTTACCGTCCTCCAGAGGGGTTGGT

Supplementary Table S3: qRT-PCR primers

Name	Sequences
Ino80-PF	TGGCTAAAGAGCATTCTGCTAAG
Ino80-PR	TGTGTAGTCGAAGCATGTTGTG
Nanog-PF	AAGGTCCCGGTCAAGAAACAG
Nanog-PR	CTTCTGCGTCACACCATTGC
p53-PF	CAGCACATGACGGAGGTTGT
p53-PR	TCATCCAAATACTCCACACGC
Bcl2-PF	GGTGGGGTCATGTGTGTGG
Bcl2-PR	CGGTTCAGGTACTCAGTCATCC
Bax-PF	CCCGAGAGGTCTTTTTCCGAG
Bax-PR	CCAGCCCATGATGGTTCTGAT
CyclinD1-PF	CAATGACCCCGCACGATTTC
CyclinD1-PR	CATGGAGGGCGGATTGGAA
Gapdh-PF	GGAGCGAGATCCCTCCAAAAT
Gapdh-PR	GGCTGTTGTCATACTTCTCATGG
CyclinE1-PF	GCCAGCCTTGGGACAATAATG
CyclinE1-PR	CTTGCACGTTGAGTTTGGGT

Supplementary Table S4: ChIP-qPCR primers

Name	Sequences
Oct4 upstream site PF	CAAAGTGCTGGGATTATGCTGG
Oct4 upstream site PR	CGACAGAGCAAGACTCCGTC
Oct4 TSS PF	TGGAGCATGGCACGTGATTCC
Oct4 TSS PR	CTGACTTCCGGAACGAACCGT
Nanog upstream site PF	TTGATCTACTCTACTAAATC
Nanog upstream site PR	AGGTTGCAGCGAGCCGAGATC
Nanog TSS PF	AGGTTGCAGCGAGCCGAGATC
Nanog TSS PR	TGTCAAGGCCACCAAGACTCA
